# Supplementary material for: Lymphocyte Population Changes at Two Time Points during the Acute Period of COVID-19 Infection
Source: J Clin Med. 2022 Jul 25;11(15):4306. doi: 10.3390/jcm11154306 (PMC9329935; doi:10.3390/jcm11154306)
Supplement: Supplementary file 1 [file jcm-11-04306-s001.zip › jcm-1792292-supplementary.pdf]

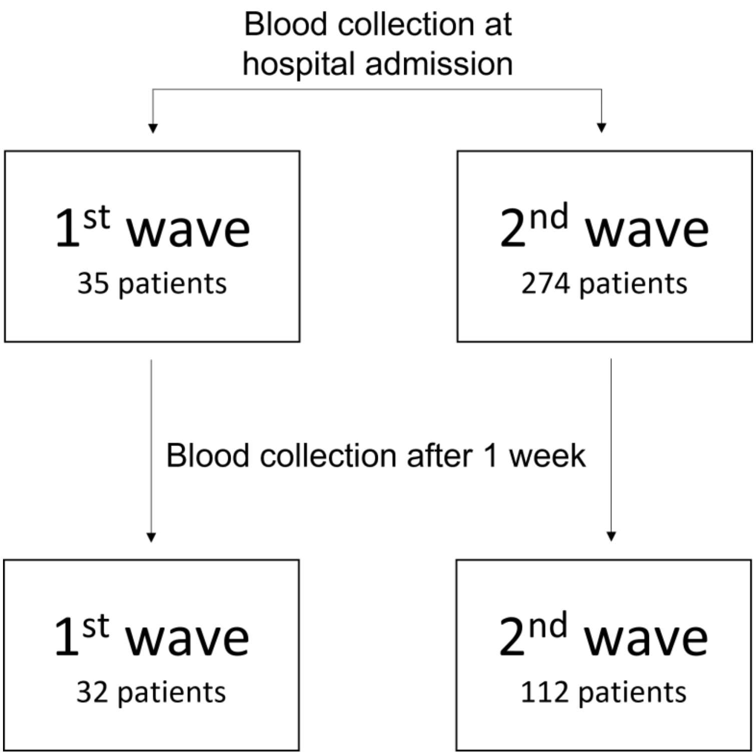

Figure S1. Flow-chart of COVID-19 patients' selection. A follow-up to 1 week has been carried out for 32 patients from the 1st COVID-19 wave and 112 patients from the 2nd COVID-19 wave.

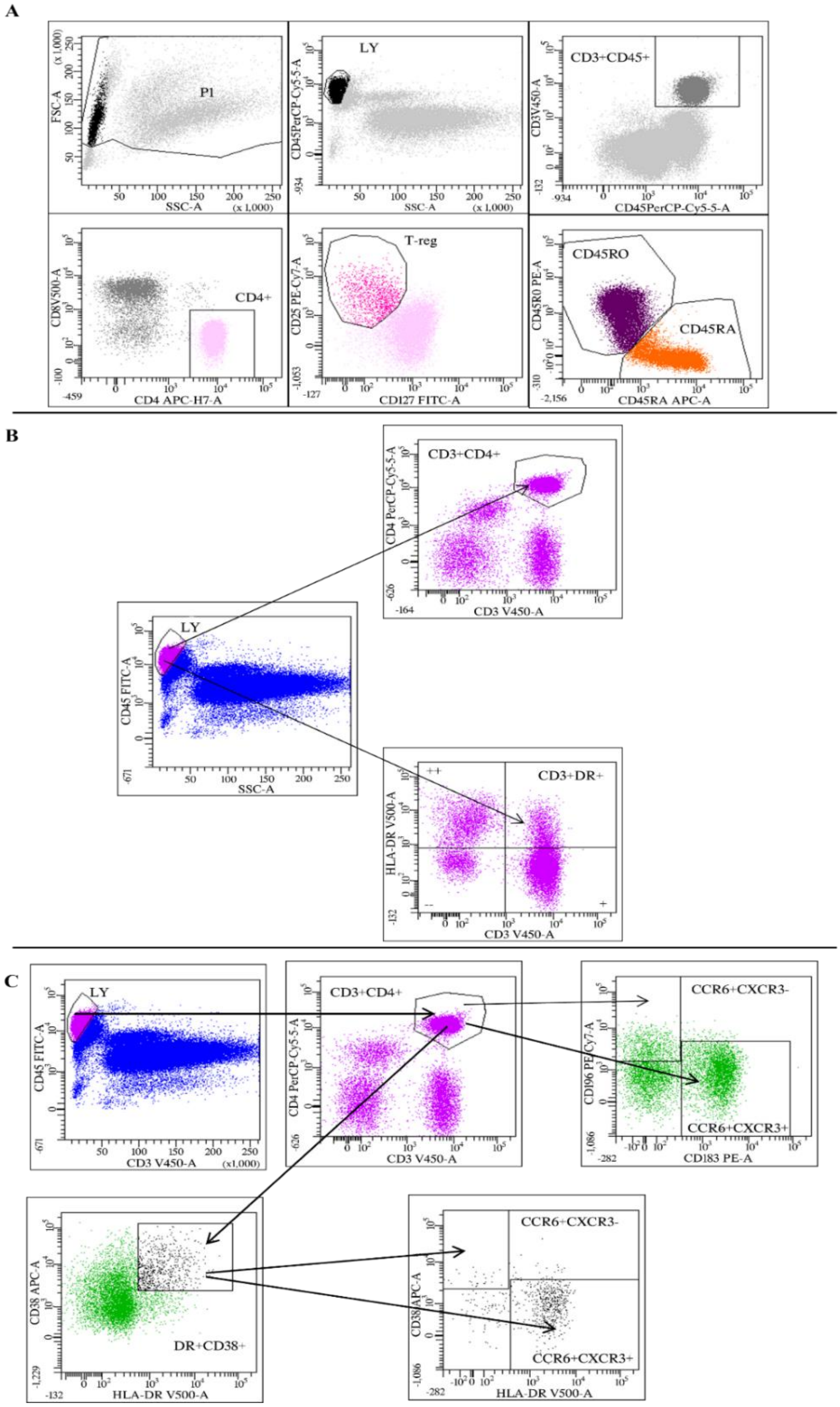

Figure S2. Lymphocyte population analysis. A) Lymphocyte cells were gated by using CD45 vs SSC-A, and this gate was used to identify T lymphocytes (CD45+, CD3+). T helper (TH) and T cytotoxic cells have been identified as CD3+, CD4+ and CD3+, CD8+, respectively. Naïve and memory cells have been identified as CD45RA+ and CD45RO+, respectively. T regulatory cells have been identified as CD3+, CD4+, CD25+, CD127-. B) From the lymphocytes (CD45 vs SSC-A), activated T lymphocytes have been identified as CD3+, DR+. C) From TH cells (CD3+, CD4+), TH17 and TH1 have been identified as CCR6+, CXCR3- and CCR6-, CXCR3+, respectively. CD38+ and HLA-DR+ were used to identify their activated form. CD19 and CD56 were used to identify B cells and Natural killer (NK) cells respectively.

Table S1. Comparison of circulating lymphocyte subpopulations in 144 COVID-19 patients with different severity at hospital admission (basal) and after 1 week. Median (interquartile range). For each parameter, we report the reference range.

		Mild (n = 29)	Moderate (n = 79)	Severe (n = 28)	Died (n = 8)
Naïve (%)	Basal	65 (59; 72)	64 (56; 72)	63 (57; 72)	49 (41; 63)
(13-61)	After 1 week	58 (53; 68)	61 (52; 69)	59 (52; 67)	67 (59; 71)
	Δ (%)	-6.8 (-16; 5.1)	-6.9 (-14; 2.8)	-9.5 (-16; -0.04)	24 (2.7; 51)
	P value	n.s.	**0.002**	**0.002**	**0.028**
Naïve (N/mmc)	Basal	655 (459; 1207)	571 (411; 977)	614 (346; 1197)	417 (251; 583)
(126 – 1121)	After 1 week	973 (681; 1492)	764 (474; 1242)	800 (497; 1142)	540 (221; 783)
	Δ (%)	29 (-0.4; 68)	27 (-27; 105)	29 (-31; 81)	27 (-56; 244)
	P value	**0.012**	**0.003**	n.s.	n.s.
NK (%)	Basal	7.0 (4.0; 13.5)	10 (5.0; 15)	9.0 (6.0; 18)	15 (11; 26)
(4-28)	After 1 week	9.0 (3.5; 13)	8.0 (5.0; 13)	8.0 (5.0; 11)	14 (3.7; 21)
	Δ (%)	0.0 (-26; 38)	-3.4 (-42; 25)	-11 (-57; 15)	-21 (-49; 40)
	P value	n.s.	**0.016**	n.s.	n.s.
NK (N/mmc)	Basal	103 (45; 196)	91 (47; 156)	89 (53; 165)	103 (85; 186)
(73 – 654)	After 1 week	156 (69; 227)	109 (54; 173)	103 (50; 174)	73 (47; 174)
	Δ (%)	48 (-7.4; 99)	16 (-21; 73)	-4.3 (-47; 73)	-49 (-68; 79)
	P value	**0.016**	n.s.	n.s.	n.s.
B (%)	Basal	11 (7.0; 16)	13 (7.0; 20)	14 (7.5; 18)	9.5 (3.0; 17)
(5-20)	After 1 week	10 (5.5; 15)	11 (6.0; 19)	13 (8.0; 21)	14 (4.2; 26)
	Δ (%)	-14 (-33; 13)	0.0 (-31; 25)	11 (0.0; 29)	45 (7.7; 66)
	P value	n.s.	n.s.	n.s.	**0.034**
B (N/mmc)	Basal	138 (66; 292)	119 (61; 226)	125 (65; 247)	47 (36; 130)
(72 – 520)	After 1 week	173 (83; 262)	127 (83; 271)	150 (82; 302)	78 (22; 340)
	Δ (%)	28 (-32; 76)	9.7 (-21; 101)	54 (-30; 99)	-18 (-50; 398)
	P value	n.s.	n.s.	**0.021**	n.s.
Memory (%)	Basal	34 (28; 41)	36 (28; 43)	37 (27; 43)	48 (36; 58)
(20-65)	After 1 week	42 (31; 48)	39 (30; 48)	40 (33; 47)	33 (29; 41)
	Δ (%)	9.4 (-12; 31)	11 (-6.4; 30)	22 (-1.5; 30)	-33 (-37; -0.4)
	P value [a]	n.s.	**0.001**	**0.004**	**0.042**
Memory (N/mmc)	Basal	477 (192; 718)	354 (202; 564)	350 (214; 509)	333 (161; 683)
(319-1184)	After 1 week	618 (425; 806)	474 (346; 651)	534 (306; 767)	281 (130; 534)
	Δ (%)	30 (4.3; 153)	39 (-5.8; 112)	46 (1.5; 176)	-41 (-67; 107)
	P value [a]	**0.001**	**< 0.0001**	**0.002**	n.s.

Paired comparisons between the data at hospital admission (basal) and after 1 week were evaluated by Wilcoxon signed-rank test. Significant values are reported in bold. n.s.: not significant.

Table S2. Comparison of activated and TH lymphocyte populations in 144 COVID-19 patients with different severity at hospital admission (basal) and after 1 week. Median (interquartile range). For each parameter we report the reference range.

		Mild (n = 29)	Moderate (n = 79)	Severe (n = 28)	Died (n = 8)
Total activated	Basal	15 (10; 22)	18 (13; 26)	17 (11; 24)	14 (7.0; 22)
(%)	After 1 week	13 (9.5; 21)	16 (12; 23)	18 (11; 25)	21 (13; 31)
(6-31)	Δ (%)	-17 (-27; 27)	-7.1 (-33; 31)	14 (-12; 32)	66 (-2.2; 100)
	P value	n.s.	n.s.	n.s.	n.s.
Total activated	Basal	170 (100; 366)	173 (101; 286)	169 (102; 281)	80 (53; 232)
(N/mmc)	After 1 week	240 (129; 405)	204 (123; 377)	184 (127; 342)	159 (43; 417)
(86 – 799)	Δ (%)	45 (-25; 97)	17 (-30; 74)	41 (-11; 103)	-1.6 (-43; 334)
	P value	n.s.	**0.020**	**0.021**	n.s.
T activated (%)	Basal	3.0 (2.0; 4.0)	3.0 (2.0; 5.0)	2.0 (1.0; 5.0)	3.0 (3.0; 3.7)
(1-18)	After 1 week	3.0 (1.5; 6.0)	4.0 (2.0; 7.0)	3.0 (1.0; 4.5)	4.5 (2.5; 8.2)
	Δ (%)	-17 (-50; 100)	0.0 (-50; 100)	0.0 (-37; 100)	57 (-19; 100)
	P value	n.s.	n.s.	n.s.	n.s.
T activated	Basal	29.6 (9.3; 44.7)	23.7 (12.9; 34.5)	15.8 (7.2; 35.7)	14.6 (9.4; 26)
(N/mmc)	After 1 week	30.6 (13.4; 75.1)	32.8 (16.6; 62.4)	23.5 (13.9; 43.4)	18.5 (11.4; 37.4)
(14 – 411)	Δ (%)	22 (-44; 220)	26 (-39; 173)	40 (-24; 232)	16 (-39; 197)
	P value	n.s.	**0.022**	n.s.	n.s.
TH1 (%)	Basal	24 (18; 28)	18 (13; 25)	20 (17; 23)	17 (16; 25)
(5-27)	After 1 week	23 (16; 29)	21 (17; 26)	21 (13; 25)	23 (7.2; 36)
	Δ (%)	0.0 (-13; 25)	15 (-5.9; 67)	4.8 (-21; 53)	16 (-52; 103)
	P value	n.s.	**0.001**	n.s.	n.s.
TH1 (N/mmc)	Basal	147 (56; 180)	70 (43; 133)	58 (35; 108)	71 (30; 132)
(37 – 220)	After 1 week	186 (99; 244)	119 (71; 195)	100 (49; 145)	49 (29; 161)
	Δ (%)	34 (-2.8; 86)	50 (-15; 155)	40 (-36; 221)	18 (-34; 61)
	P value	**0.004**	**< 0.0001**	n.s.	n.s.
TH17 (%)	Basal	9.0 (7.0; 15)	11 (6.0; 17)	8.0 (6.0; 12)	9.5 (3.5; 13)
(1.7-19.3)	After 1 week	9.0 (6.0; 12)	10 (6.0; 14)	11 (8.0; 14)	7.0 (3.6; 13)
	Δ (%)	0.0 (-29; 21)	-14 (-46; 43)	37 (-21; 115)	-29 (-47; 18)
	P value	n.s.	n.s.	n.s.	n.s.
TH17 (N/mmc)	Basal	49 (29; 66)	43 (25; 71)	31 (19; 66)	29 (12; 78)
(3.8 – 60.0)	After 1 week	70 (43; 114)	48 (25; 92)	55 (38; 81)	27 (8.5; 44)
	Δ (%)	38 (-91; 89)	1.6 (-45; 164)	70 (4.8; 403)	-0.7 (-61; 39)
	P value	n.s.	n.s.	**0.001**	n.s.

Paired comparisons between the data at hospital admission (basal) and after 1 week were evaluated by Wilcoxon signed-rank test. Significant values are reported in bold. n.s.: not significant.
